# Targeting Evolutionary Conserved Oxidative Stress and Immunometabolic Pathways for the Treatment of Respiratory Infectious Diseases

**DOI:** 10.1089/ars.2020.8028

**Published:** 2020-03-24

**Authors:** Jonathan R. Erlich, Eunice E. To, Stella Liong, Robert Brooks, Ross Vlahos, John J. O'Leary, Doug A. Brooks, Stavros Selemidis

**Affiliations:** ^1^ Program in Chronic Infectious and Inflammatory Diseases, Oxidant and Inflammation Biology Group, School of Health and Biomedical Sciences, College of Science, Engineering & Health, RMIT University, Bundoora, Australia.; ^2^ School of Pharmacy and Medical Sciences, University of South Australia Cancer Research Institute, University of South Australia, Adelaide, Australia.; ^3^ Department of Histopathology, Trinity College Dublin, Dublin, Ireland.; ^4^ Sir Patrick Dun's Laboratory, Central Pathology Laboratory, St James's Hospital, Dublin, Ireland.; ^5^ Molecular Pathology Laboratory, Coombe Women and Infants' University Hospital, Dublin, Ireland.

**Keywords:** metabolism, NADPH oxidase, mitochondria, influenza, immunity, co-infection

## Abstract

***Significance:*** Up until recently, metabolism has scarcely been referenced in terms of immunology. However, emerging evidence has shown that immune cells undergo an adaptation of metabolic processes, known as the metabolic switch. This switch is key to the activation, and sustained inflammatory phenotype in immune cells, which includes the production of cytokines and reactive oxygen species (ROS) that underpin infectious diseases, respiratory and cardiovascular disease, neurodegenerative disease, as well as cancer.

***Recent Advances:*** There is a burgeoning body of evidence that immunometabolism and redox biology drive infectious diseases. For example, influenza A virus (IAV) utilizes endogenous ROS production *via* NADPH oxidase (NOX)2-containing NOXs and mitochondria to circumvent antiviral responses. These evolutionary conserved processes are promoted by glycolysis, the pentose phosphate pathway, and the tricarboxylic acid (TCA) cycle that drive inflammation. Such metabolic products involve succinate, which stimulates inflammation through ROS-dependent stabilization of hypoxia-inducible factor-1α, promoting interleukin-1β production by the inflammasome. In addition, itaconate has recently gained significant attention for its role as an anti-inflammatory and antioxidant metabolite of the TCA cycle.

***Critical Issues:*** The molecular mechanisms by which immunometabolism and ROS promote viral and bacterial pathology are largely unknown. This review will provide an overview of the current paradigms with an emphasis on the roles of immunometabolism and ROS in the context of IAV infection and secondary complications due to bacterial infection such as *Streptococcus pneumoniae*.

***Future Directions:*** Molecular targets based on metabolic cell processes and ROS generation may provide novel and effective therapeutic strategies for IAV and associated bacterial superinfections.

## Introduction

The rise of photosynthesis by certain ancient cyanobacteria posed a major challenge to existing life forms, which had to survive a reducing atmosphere, but largely drove the evolution of eukaryotes. The release and the substantial elevation in atmospheric oxygen concentrations termed the “great oxygenation event” initiated enormous evolutionary pressures on life forms to deal with a hostile oxidizing environment. On one side, the availability of oxygen was beneficial for multiple reasons. It allowed for a greater capacity and efficiency in energy generation from carbon-based fuels than the most efficient anaerobic pathways, owing to its ground-state allotrope, triplet oxygen being stable and thus allowing for accumulation in the atmosphere. On the other hand, oxygen is the precursor to a multitude of reactive oxygen species (ROS) that have the capacity to cause damage to cellular macromolecules such as DNA, proteins, and lipids. However, despite ROS having the capacity to damage DNA, an evolutionary adaptation might have been sexual reproduction. Some early forms of prokaryotes would have been exposed to high levels of oxygen, and as such adapted new means for bacterial transformation (*i.e.*, the transfer of DNA from one bacterium to another) giving rise to eukaryotic sexual reproduction. Indeed, sexual reproduction or meiosis was probably facilitated as a consequence of DNA damage arising from oxidizing stressful conditions in the surrounding environment. Alternatively, cells also evolved a series of biological molecules and proteins to deal with the consequences of living in an oxidizing atmosphere, including powerful antioxidant enzymes. Indeed, the first such enzymes were superoxide dismutases (SODs), catalase, glutathione reductase, and cellular glutathione—a fine balance of ROS production and removal is critical for survival. All aerobic prokaryotes, including *Escherichia coli* and other bacteria, as well as eukaryotes express these antioxidants. There is no doubt that ROS are ancient molecules that were pivotal for the evolution of life as we know it. Hence, it is not surprising that ROS pervade all biological processes from physiological aerobic energy metabolism processes to a wide variety of pathological diseases caused by infectious agents such as bacteria and viruses, which change the dynamic balance of ROS production. This review focuses on the metabolic and oxidative stress pathways activated by bacteria and viruses; there is no doubt that co-evolution of these pathogens coincides with the initial oxygenation events that occurred more than ∼2.5 billion years and, as such, adapted to utilizing ROS for their advantage. This review is primarily focused on respiratory infectious viruses and bacteria—indeed, the motivation for this stems from the fact that the lung is the primary site of oxygen metabolism and also the primary site of infection of certain viruses such as influenza A virus (IAV) and bacteria such as *Streptococcus pneumoniae*. We also summarize novel pharmacotherapeutics focused on exploiting immunometabolism and oxidative stress with an emphasis on how these might guide the treatment of respiratory infectious diseases such as those caused by IAV and *S. pneumoniae*.

## Immunometabolism

Historically, the mitochondria have been considered to function as the powerhouse of the cell, with their primary function to generate energy, mainly in the form of adenosine triphosphate (ATP) through oxidative phosphorylation. Indeed, between 1948 and 1951, mitochondria were identified as the site for housing enzymes belonging to the tricarboxylic acid (TCA) cycle, oxidative phosphorylation, and fatty acid oxidation (73, 147). Since then, major discoveries have significantly advanced our understanding of how the mitochondria function, from the first discovery that the mitochondria are capable of producing ROS (11), to more novel discoveries, which show that changes in metabolic function govern immune cell function during acute and chronic inflammation in respiratory diseases, cancer, and metabolic syndromes.

It was once believed that metabolism was merely a process for energy production through ATP. Aside from metabolic diseases such as obesity and diabetes, historically, there has been relatively little interest in studying changes in metabolic processes that occur during acute and chronic inflammation. Many previous studies examined the energy expenditure as an output in relation to inflammation; consequently, as immune cells became activated and differentiated, the cells' energy demands would increase, thus necessitating increased metabolic output. However, over the past few years, the traditional view of metabolism has changed, and ultimately there is a need for a new understanding of how metabolism and key metabolites influence the inflammatory state. In this review, we will primarily focus on glycolysis, the pentose phosphate pathway (PPP), and the TCA cycle. The changes in these metabolic pathways not only form critical products involved in the inflammatory process, as the intermediate metabolites can both directly and indirectly modulate the production of ROS, which they themselves are able to contribute to the pathogenesis of multiple diseases, including respiratory tract infections.

## Glycolysis

The universal use of glycolysis by organisms underlies its role as an ancient metabolic pathway that is responsible for the generation of energy. Glycolysis is a 10-step enzyme-catalyzed process that occurs in the cytosol. Glycolysis is relatively inefficient, and it can be either anaerobic, resulting in the formation of lactic acid, or aerobic, which results in the formation of pyruvate. Both aerobic and anaerobic glycolysis results in two ATP molecules per glucose molecule. Intermediate products generally feed into other metabolic pathways, such as glucose-6-phosphate into the PPP, and in the case of aerobic glycolysis: pyruvate, which can be converted into acetyl CoA to enter the TCA cycle. One of the products of glycolysis is NADH, an efficient reducing agent and cofactor for many enzymes. Glycolysis also provides vital intermediates for the synthesis of ribose, fatty acids, and amino acids. Proliferating cells will upregulate glycolysis as their dominant metabolic pathway (89), and this is particularly evident in immune cells responding to damage or pathogens. Indeed, bacterial lipopolysaccharide (LPS) is detected by toll-like receptor (TLR)4 and can activate macrophages and dendritic cells in a glycolytic-dependent manner (162). The activation of the glycolytic pathway is highly dependent on phosphoinositide 3-kinase (PI3K)/protein kinase B (AKT) activation, as inhibitors of PI3K can reduce the glycolytic rate of LPS-stimulated dendritic cells back to baseline (78). In addition, Krawczyk *et al.* observed a suppression of 5′ adenosine monophosphate-activated protein kinase (AMPK) phosphorylation after LPS stimulation, and the utilization of small molecular activators of AMPK suppressed the LPS-induced production of interleukin (IL)-12p40, whereas inhibitors potentiated this effect (78). As AMPK phosphorylation is critical for mediating oxidative phosphorylation, there appears to be an intricate negative feedback mechanism occurring between glycolysis and oxidative phosphorylation (122).

**FIG. 1.**
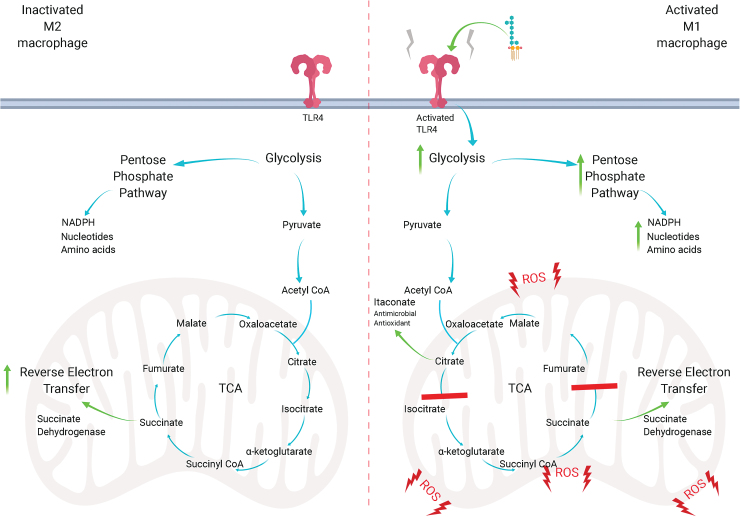
**The metabolic switch.** On activation by LPS, macrophages become activated, causing increases in glycolysis, which allows for rapid generation of ATP. The PPP is also upregulated, which allows for the increased generation of NADPH, nucleotides, and amino acids to assist in proliferation. The TCA cycle is broken into two parts of the cycle, after citrate, which allows the formation of the antimicrobial itaconate, and succinate, which fuels the electron transfer chain. Accumulation of succinate causes a change from oxidative phosphorylation to reverse electron transfer, increasing the leakage of electrons and generation of mitochondrial ROS. ATP, adenosine triphosphate; LPS, lipopolysaccharide; NADPH, nicotinamide adenine dinucleotide phosphate; PPP, pentose phosphate pathway; ROS, reactive oxygen species; TCA, tricarboxylic acid. Color images are available online.

Early work demonstrated that activated macrophages and T cells undergo a metabolic switch from oxidative phosphorylation to glycolysis (Fig. 1). Using 2-deoxyglucose to suppress hexokinase, and hence glycolysis, suppressed macrophage activation and subsequent inflammatory processes in *in vivo* models (108). It is likely that the increased metabolic demand, due to rapid proliferation, is a primary cause for the switch to glycolysis (Fig. 1). Notably, LPS-dependent activation of macrophages leads to an enhanced expression of hypoxia-inducible factor-1α (HIF-1α), which transcribes key enzymes in the glycolytic pathway (162). In addition, LPS can form a complex with pyruvate kinase isoenzyme M2, which is able to promote HIF-1α-dependent genes, such as the proinflammatory cytokine IL-1β (122, 128). Aside from inflammation, hypoxia drives HIF-1α activation, preventing the hydroxylation of proline residues, which, under normoxic conditions, would target HIF-1α to be degraded by the proteasome, as oxygen is an important co-substrate for HIF protyl-hydroxylases (97, 152). Inhibition of HIF-1α promotes the M2 phenotype of macrophages over M1, signaling its important role in inflammation (128).

## Pentose Phosphate Pathway

In mammalian cells, the PPP runs parallel to glycolysis within the cytosol, and it is interconnected with the glycolysis pathway through glucose-6-phosphate, fructose-6-phosphate, and glyceraldehyde-3-phosphate. The products of the PPP include ribose 5-phosphate and erythrose 4-phosphate, which are used in the synthesis of nucleotides and amino acids, respectively. In this sense, PPP aids in protein production, cell survival, and proliferation (30).

During inflammation, the PPP is upregulated, and it is most likely to generate the NADPH, amino acids, and nucleotides, which are necessary to support immune cell activation and proliferation (122). NADPH reduces glutathione disulfide in the presence of glutathione reductase, forming NADP+ and two glutathione (GSH) molecules. Conversely, NADPH can be utilized as the electron donor to fuel the NADPH oxidase (NOX) oxidases, such as NOX2, which generates the oxidative burst that helps in bacterial clearance (55). What is not yet established is the crosstalk between the NOX oxidases and GSH, and how this is used to control the dynamic balance of ROS (122). Due to the highly reactive nature of ROS, it is likely that these pro- and antioxidant systems derived from the PPP are compartmentalized and, therefore, do not necessarily crosstalk directly, but rather this might be mediated through secondary signaling pathways.

The generation of ribose-5-phosphate from the PPP allows for the biosynthesis of not only the nucleotides but also the nucleic acids involved in DNA and RNA generation. This increase in DNA and RNA supports the proliferation of immune cells and the response required to clear an infection. Importantly, this increase in DNA supports the generation of neutrophilic extracellular traps (NETs) that aid in the clearance of pathogens (6). An excess of DNA, however, might be responsible for an increase in pathogenic burden. For example, multiple studies have associated a reduction in neutrophils, and by association the NETs, with a reduction in lung pathology in response to influenza infection (167, 169, 176). This is particularly important as influenza infections typically result in an exacerbated immune response. In addition, the virus itself likely favors an increase in the PPP, as the nucleotides themselves can provide the building blocks for viral replication.

## TCA Cycle

The TCA cycle (otherwise known as the citric acid cycle or Krebs cycle) is a major metabolic pathway that, along with oxidative phosphorylation, is very efficient at energy generation. It is the preferred form of energy production in quiescent and non-proliferating cells, as well as cells that are generally long-lived such as memory CD8+ T cells (122). The TCA cycle utilizes metabolites at multiple points to fuel this metabolic process. Pyruvate generated *via* glycolysis and fatty acids can be converted to acetyl CoA, which is then converted to citric acid. In addition, glutamate is a critical amino acid that enters the TCA cycle and is converted to α-ketoglutarate. NADH and FADH_2_ are produced through the TCA cycle, which interconnects with oxidative phosphorylation at succinate dehydrogenase (SDH, also known as mitochondrial complex II).

Rapid cell proliferation and growth downregulate the TCA cycle, as amino acids and fatty acids are diverted to promote cell proliferation. Thus, an inflammatory M1 macrophage displays a reduction in the TCA cycle and an increase in glycolysis, and, in fact, the TCA cycle is broken twice: after citrate (103) and succinate (162). Citric acid can be transported from the mitochondria into the cytosol, where it can be used for fatty acid generation and membrane synthesis. It can also be used to generate prostaglandins, effector molecules of macrophages that are also pro-inflammatory (121). Citrate can be used to generate itaconate, a metabolite that has potent anti-bactericidal effects (107) and anti-inflammatory and antioxidant effects *via* its activation of anti-inflammatory transcription factors such as nuclear erythroid factor 2 (NRF2) (110). Itaconate is generated by citric acid through the enzyme immune responsive gene 1 (IRG1), which generates *cis*-aconitate decarboxylase. Itaconate has multiple anti-inflammatory, antioxidant, and antimicrobial properties that make it an exciting therapeutic. Indeed, exogenous itaconate can lead to an increased accumulation of succinate, but labeled itaconate is not observed in succinate and instead there are reductions in SDH activity (21). Itaconate can also exert its antioxidant effects by alkylation of kelch-like ECH-associated protein 1 (KEAP1) cysteine residues, which would otherwise rapidly degrade NRF2. Alkylation of KEAP1 allows NRF2 to migrate to the nucleus where it can begin to activate antioxidant and anti-inflammatory gene transcription, including heme oxygenase 1 and GSH (51, 110).

Accumulation of succinate, on the other hand, was shown to stabilize HIF-1α, promoting glycolysis and production of the pro-inflammatory cytokine IL-1β (162). Another consequence of succinate accumulation is mitochondrial ROS (mtROS) production. SDH, which not only oxidizes succinate to fumarate but also participates in the electron transport chain (126), was, indeed, reported to be a main driver of mtROS (109). Accumulation of succinate drives SDH, which can drive mitochondrial complex I through reverse electron transfer (RET), increasing the leakage of electrons to molecular oxygen (109), resulting in superoxide generation (Fig. 1).

## Reactive Oxygen Species

Originally believed to only occur as by-products of normal cellular respiration, ROS are now understood to be molecules with physiological functions and mediators of cardiovascular disease (CVD), cancers, and infectious diseases (63, 86, 130). These molecules are generally short-lived, in part due to their highly reactive nature, as well as dedicated endogenous antioxidant systems that neutralize them. ROS are formed when molecular oxygen is reduced by electrons to form superoxide. Superoxide is converted to hydrogen peroxide (H_2_O_2_) *via* SOD and then to water and oxygen through either catalase or glutathione peroxidase (Gpx). The reactivity of superoxide and H_2_O_2_ is quite limited; superoxide preferentially targets proteins containing iron clusters, whereas H_2_O_2_ targets cysteine residues (175). This makes it especially dangerous due to the Fenton reaction, which causes the conversion of H_2_O_2_ to two hydroxyl radicals through the oxidation of iron, which are permeable to cell membranes and can react with almost all biological molecules, thus making it highly reactive (174). Another highly reactive molecule is peroxynitrite, which is formed by the reaction of nitric oxide with superoxide and is considered among the fastest known biological reactions. With no known catalyst, this reaction between nitric oxide (NO) and superoxide is only limited by the amount of substrate that is available (127). In general, ROS are kept at low levels and are involved in a range of functions such as cofactors for proteins and redox signaling. High levels of ROS can cause protein degradation *via* post-translational modification, lipid oxidation, and DNA damage, all of which fall under the umbrella term “oxidative stress” (32).

## Mitochondrial ROS

As described earlier, a metabolic switch to glycolysis from oxidative phosphorylation results in mtROS production *via* the electron transport chain (2, 4, 113). mtROS is formed by the leakage of electrons from mitochondrial complexes I and III and taken up by oxygen to form superoxide. Once believed to be merely a harmful by-product of normal cellular respiration, it is now understood to have many important biological functions, including post-translational protein modification (153). At low levels, mtROS is understood to facilitate adaptations to hypoxic conditions. Indeed, mtROS is required for the stabilization of HIF-1α mRNA (40). TLR4 and TLR2 activation also leads to a heightened mtROS response, which, in turn, regulates key inflammatory processes. HIF-1α, as described earlier, leads to the upregulation of glycolytic genes (179). Redox regulation of the inflammasome nod-like receptor protein 3 (NLRP3) also occurs through mtROS, likely through modification of conserved cysteine residues (1, 36). As inflammasome activation leads to the generation of powerful inflammatory cytokines such as IL-1β and IL-18, mtROS regulate inflammation. However, due to the proximity, mtROS might also readily attack nuclear and mitochondrial DNA, which has been implicated in neurodegenerative disorders, aging, and cancer (158). Due to the sensitivity of DNA to oxidative damage, mitochondria employ multiple, powerful antioxidants to neutralize ROS such as SOD enzymes and GSH.

SODs are a class of enzymes that are responsible for the catalysis of superoxide to H_2_O_2_. Three forms of SOD are known to exist in humans, with SOD2 being the predominant class within the mitochondria. SOD2 tightly regulates ROS-induced apoptosis; indeed, knockout cell lines of SOD2 were more prone to cell death compared with wild type, and SOD2-overexpressing cells were the most protected (68, 79). Although this system would be protective against cardiovascular and neurodegenerative disorders, it could prove to be problematic in the regulation of cancer cells due to its protection against cell death (39, 79, 184). SOD2 also appears to be a critical regulator of the innate immune response; SOD2 knockout zebra fish embryos had a significantly increased bacterial load at 20-h post-infection compared with wild-type controls. The SOD2 knockout bacterial load was restored to wild-type controls when the mtROS scavenger MitoTEMPO was administered (134).

GSH is an antioxidant that reacts with free radicals and neutralizes them. Once in its oxidized form, it can be reduced and recycled through glutathione reductase (22). The reaction between GSH and H_2_O_2_ is catalyzed by the enzyme family Gpx, which also reduces lipid hydroperoxides. Gpx-1, in particular, has been implicated in chronic obstructive pulmonary disease (COPD) and other inflammatory diseases (174). The global lung expression of Gpx-1 has been found to be significantly decreased after smoke exposure (10). Indeed, Gpx-1 knockout mice showed significant elevations in inflammatory neutrophils, macrophages, IL-17, and macrophage inflammatory protein 1α compared with their wild-type counterparts in COPD models. Treatment with ebselen, a Gpx-1 mimetic, not only reversed this phenotype but also improved overall pathology of the wild-type mice (34). Similarly, Gpx-1 plays an anti-inflammatory role in influenza A infections (IAV) (182). Infiltrating immune cells in the bronchoalveolar lavage fluid (BALF) were significantly increased in IAV-infected Gpx-1 knockout mice, compared with their infected wild-type counterparts. Ebselen was able to significantly reverse this phenotype, comparable to the wild-type controls (182). Not only does this highlight the anti-inflammatory effects of Gpx-1, but also the pathological role that H_2_O_2_ plays during disease. On its own, GSH itself also has antimicrobial properties. NO is able to inhibit bacterial growth in the case of *Mycobacterium tuberculosis* and *Salmonella typhimurium,* but it is limited by its short half-life (19, 20, 112). NO can combine with GSH, which stabilizes its half-life and, thus, becomes more efficient for bacterial inhibition, by releasing NO to kill the pathogen (9, 27, 117). GSH is also implicated in cancer, as a potential mechanism for chemotherapy resistance. Indeed, it offers protection against mitochondrial DNA damage, which would otherwise be induced by chemotherapy, thus promoting tumor survival in an otherwise hostile environment (180).

### NADPH oxidase

The most conclusive evidence for ROS having important biological functions is the presence and conservation of NOX and dual oxidase (DUOX) enzymes, which are dedicated ROS-generating enzymes. The defining feature of the NOX/DUOX family is that they use NADPH as an electron donor, which transfers electrons *via* the transport chain using FAD and heme moieties, ultimately resulting in oxygen being the electron acceptor (32). In NOX1, NOX2, and NOX5, the final product is superoxide, whereas NOX4, DUOX1, and DUOX2 lead to the direct production of H_2_O_2_ (32, 140).

NOX4 has diverse actions, some protective and some detrimental. For instance, NOX4 promotes endothelial cell survival, proliferation, and migration that are critical for early angiogenesis, but it has also been implicated in endoplasmic reticulum-dependant oxidative stress, oxidative DNA damage, apoptosis, and necrosis (32). Surprisingly, NOX1, located in lung epithelial cells, has also been shown to have protective effects in reducing lung injury; however, the mechanism remains largely unknown (150). Evidence suggests that H_2_O_2_ leads to increased expression of nuclear factor kappa B (NF-κB), the cytokine responsible for increased inflammatory gene expression (175). Thus, manipulation of NOX oxidases, in particular those involved in the pathogenesis of influenza, could prove vital in the development of therapeutics against inflammatory diseases.

### NOX2 oxidase

NOX2 oxidase is best characterized by its presence in phagocytes, playing a key role in neutralizing bacterial and fungal pathogens through oxidation. Deficiencies in this enzyme is one of the causes of chronic granulomatous disease, in which normally benign pathogens can cause serious morbidity and mortality (54, 173).

Evidence shows that ROS derived from NOX2 play a critical role in the lung injury and inflammation caused by IAV in mice. In fact, NOX2 appears to be the primary source of ROS generation. Using a mouse-adapted Hong Kong strain of IAV (Hk X-31), there was a significant increase in ROS generation in wild-type mice, compared with an NOX2^−/y^, which produced little, if any, ROS by their inflammatory cells (176). Interestingly, endosomal NOX2 generated ROS is a powerful regulator of the inflammatory response during an influenza infection and can promote the pathogenic process. Indeed, after Hk X-31 influenza infection, NOX2^−/y^ mice showed significantly reduced inflammation compared with the response seen in wild-type mice (176). Of note, NOX2^−/y^ mice displayed ∼40% less viral titers than wild-type mice, suggesting that ROS promote viral survival and replication (176).

NOX2 promotes CVD, including hypertension, ischemia**–**reperfusion injury after myocardial infarction, atherosclerosis, and stroke (5). Oxidative stress is a key feature in CVD, causing remodeling of the vascular system, including induction of endothelial cell apoptosis, migration of smooth muscle cells, upregulation of adhesion molecules, and lipid oxidation (129, 177). To demonstrate the importance of NOX2 in CVD, a study using NOX2-deficient mice showed reductions in oxidative stress, downregulation of the mitogen-activated protein kinase (MAPK) signaling pathway that is responsible for cardiovascular remodeling, and protection against advanced heart failure (131). Within the brain, angiotensin II can cause reperfusion in an NOX2-derived superoxide dependent manner, thus implicating NOX2 in stroke. This oxidative stress is capable of remodeling the vasculature, which can largely be responsible for the comorbidities associated with chronic inflammation such as those observed in COPD (13). Overactivity of NOX2 oxidase has been implicated in multiple cancers (61, 142). NOX2-derived ROS are known to be cytotoxic and a mutagen, which can injure healthy cells, thereby eliminating local competition for resources (170). Pharmacological inhibition of NOX2 saw reductions and regression of tumor growth in 9-day-old chicken embryos, signifying the link between tumor development and NOX2 (42). Indeed, silencing of NOX2 decreases the phosphorylation of the vascular endothelial growth factor (VEGF) receptor, greatly reducing the potential for angiogenesis and metastasis (75). This relationship is further exemplified by the co-localization of NOX2 and the VEGF receptor subtype VEGFR2 to the endosome. Murine NOX2 knockout models showed a significant reduction in tumor weights and angiogenesis and, in addition, VEGFR2 activation caused a significant increase in endosomal ROS generation (50).

## Mitochondrial and NOX2 Communication

Communication between mitochondrial-derived ROS and NOX enzymes has been reported, but the underlying mechanisms remain poorly understood. It is likely that the NADPH derived from the PPP is a key player in communication. Indeed, reduction of NADP^+^ to NADPH in the mitochondria allows the transport of electrons toward the NOX oxidases to generate ROS (32, 83). As previously mentioned, increased PPP due to the inflammatory status of macrophages generating NADPH, as well as SDH activity driving mtROS, likely provides the cocktail necessary for the induction of an NOX2-dependant oxidative burst, which, though necessary for pathogen clearance, causes great collateral damage (*e.g.*, lung injury). Redox signaling crosstalk has been extensively reviewed in Refs. (23, 24, 31, 148), therefore here we will focus on novel mechanisms of signaling between mitochondrial and NOX2 oxidase.

**FIG. 2.**
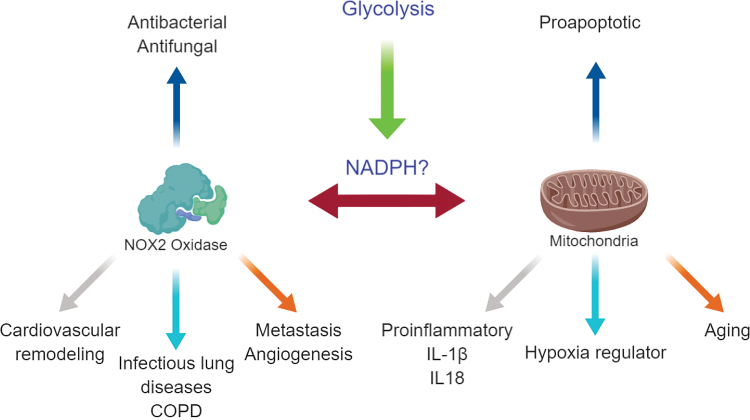
**Compartmentalization of ROS plays differing roles yet are known to communicate**. A potential mechanism is the generation of NADPH, which is controlled by increased glycolysis on macrophage activation. The NADP+ is able to accept electrons from mitochondrial-derived superoxide and it uses it to fuel the NOX oxidases. NOX, NADPH oxidase. Color images are available online.

As mtROS is dependent on multiple upstream factors such as the metabolic switch, it is possible that communication between NOX2 and the mitochondria has a common upstream signaling pathway in glycolysis. Even in the proposed model as shown in Figure 2, the amount of available NADPH is dependent on the induction of the PPP, in which glycolysis has specific substrate inputs. NADPH may not solely be responsible for this communication; there are multiple pro-inflammatory signals that could cause an increase in the activity of NOX2 oxidase. Such mediators could include the NLRP3 inflammasome complex, or a product such as IL-1β. Other potential candidates are type I interferons (IFNs) such as IFN-β. Through inhibition of TLR7, NOX2 and IFN-β are already known to be a part of an inhibitory feedback loop (46, 169). Therefore, this could be a potential mechanism by which IFN-β self-regulates to prevent autoimmunity.

## Lower Respiratory Tract Infections

Lower respiratory tract infections are the most deadly communicable disease globally and overall the third leading cause of death, causing more than 3 million fatalities in 2015 (WHO, 2018 statistics). Of these, IAV infections remain a substantial burden despite the available prevention, intervention, and prophylactic treatments. According to the World Health Organization, seasonal influenza amounts to 3–5 million cases per year; of these, up to 690,000 results in death (WHO, 2018 statistics). In addition, the economic burden remains substantial, with latest estimates costing the U.S. economy between 70 and 166 billion USD (105). In addition, high mutation rates of IAV may give rise to highly pathogenic, pandemic-causing strains, the foremost example being the 1918 H1N1 Spanish flu, notoriously being the largest IAV pandemic in history, causing around 100 million deaths (66). High amounts of transmission are generally due to a rise in population densities and, coupled with increased aviation and inadequate quarantining procedures there can be devastating consequences, as seen during the 2009 Swine flu epidemic.

Typically, symptoms of influenza are self-limiting, and they include fever, muscle aches, headaches, rhinitis, and chills. However, in susceptible populations, such as the very young, the elderly, the immunocompromised, and pregnant women, infections can result in death, primarily due to pneumonia-related complications (138). Pneumonia generally occurs after a secondary bacterial infection, whether it be due to IAV disrupting the asymptomatic flora already present or making the host more susceptible by disrupting the function of the immune system itself.

## Influenza A Virus

Despite a lack of data in regard to the metabolic properties of immune cells in response to influenza infections, the pathogenesis of the disease implies a metabolic switch is involved. Viral replication requires large amounts of energy by the cell, which is highly dependent on host metabolism (156). Indeed, the upregulated glycolytic pathway is further implicated in promoting viral pathogenicity, as observed in patients with diabetes mellitus being more susceptible to influenza infection (58). In addition, metformin, a therapeutic that is currently utilized in type II diabetes, has antiviral and anti-inflammatory properties by an unknown mechanism (41). Taken together, it can be postulated that metformin's activity against influenza A is *via* the modulation of the glycolytic pathway, by reducing the amount of available glucose.

As discussed earlier, the upregulation of the PPP can also provide an avenue for increased viral replication through increased nucleotide production. NADPH itself can be utilized to fuel NOX2 oxidase, increasing the pathology and lung injury in response to influenza (169, 176). The accumulation of both succinate and citrate within the TCA cycle could also provide an avenue for pathogenesis of influenza. In addition, accumulation of succinate causes increased stabilization, and therefore increased production of inflammatory genes such as IL-1β, potentially leading to an uncontrolled and exacerbated immune response (81, 162). Succinate also provides an avenue for increased mtROS response, which in itself is pathogenic during influenza by upregulating pro-inflammatory IL-1β, in turn downregulating the expression of the antiviral IFN-β (99). Citrate itself has the potential to both suppress and enhance viral pathogenicity. Itaconate, as described earlier, can have anti-inflammatory and antioxidant properties *via* the activation of NRF2 (110). Conversely, citrate catalyzes key steps in the formation of cholesterol through fatty acid synthesis, with cholesterols themselves having immunosuppressive properties (60). In one study, depletion of cholesterols reduced the infectivity and virion stability, whereas exogenous cholesterol reversed these phenotypic changes (8). Taken together, a wide range of targets can be identified in the metabolic pathways that are generic in nature and thus likely to not be susceptible to resistance.

### Streptococcus pneumoniae

*Streptococcus pneumoniae* are gram-positive, diplococci bacterium that are non-motile and unable to form spores. Typically, *S. pneumoniae* asymptomatically colonize the respiratory tract, sinuses, and the nasal cavity. However, pathogenicity may arise and spread to normally *S. pneumoniae* free areas in the body, commonly in susceptible populations as described earlier. *S. pneumoniae* is the most common cause of community-acquired pneumonia and meningitis (172). *S. pneumoniae* employs multiple virulence factors that aid in its ability to infect and cause disease within the host, the most significant of which is the polysaccharide capsule that envelops the bacterial cell wall. The capsule effectively “hides” the bacterial antigens while simultaneously hindering access to phagocytic cells tagged by the complement system; specifically, it inhibits C3b from effectively opsonizing *S. pneumoniae* (62). Classification of *S. pneumoniae* occurs through serotyping the capsule, with more than 90 unique serotypes having been identified, of which several have strong genetic relationships. Thus, the unique serotypes have been denoted as a number; whereas close relationships that are differentiated with letters, that is 6A and 6B, are closely related, however 6A and 9A are not.

## Influenza A and *S. pneumoniae* Co-Infection

*S. pneumoniae* are the most common bacterial infection after IAV challenge. This co-infection is particularly lethal; however, the mechanisms behind this exacerbation are poorly understood. In fact, increased bacterial load and decreased bacterial clearance have been observed in mice when co-infected with influenza, compared with SP alone (178). Multiple mechanisms have been proposed as to how the strong synergistic pathology of this co-infection is established. One of the potential mechanisms of pathogenicity involves IAV causing disruptive damage to the alveolar epithelial cells, resulting in surface membranes being exposed for the bacteria to infect (69). IAV is also able to decrease the efficiency of which the mucosa can clear the bacterial pathogen, and together with the inability for the epithelium to re-proliferate and repair itself due to infection can result in the high lethality (101). Damage to the epithelium indirectly leads to enhanced bacterial adherence through exposed host receptors and modified proteins. An example would be glycanson (101) and altered platelet activating factor receptor, all of which enhance bacterial pathogenicity (118). Indeed, *S. pneumoniae* can employ virulence factors pneumococcal surface protein A (PspA) and pneumococcal serine-rich repeat protein (PsrP). PsrP has been described as having a role in the development of biofilms (145), and it is a lung-specific adherin. PspA, alternatively, mediates the immunosuppressive property of phosphorylcholine *via* inhibition of immunoglobulin G (146).

The precise mechanism of why IAV accentuates *S. pneumoniae* infection remains poorly understood. A study by Reddinger *et al.* suggests that the viral infection inflicts physiological changes, such as release of nutrients, ATP availability, and changes in temperature that facilitate biofilm dispersal and colonization into other areas *in vitro* (137). Indeed, when infected with IAV post *Staphylococcus aureus* administration, mice were reported to have increased bacterial load in the lung compared with mice uninfected with IAV (137). However, it is unknown whether the phosphate-buffered saline solution may have simply washed any loose bacterial cells further down the respiratory system to cause further infection. Exacerbated infection may also be caused by IAV-induced destruction of the cilial and secretory trachea cells leading to the exposure of the membrane, facilitating colonization (135). Indeed, it may also be caused by epithelial damage, exposing attachment sites for *S. aureus* (154). Another potential candidate mechanism involves the impairment of the innate immune response by IAV to allow uncontrolled proliferation of the bacterium. Two possibilities exist, in which the immune response to a secondary stimulus is significantly enhanced, causing uncontrolled collateral pathology, also known as trained immunity. The other possibility is the opposite, whereby the immune cell is tolerized, causing a lack of a response to the secondary stimuli. Trained immunity and immune tolerance are discussed next.

## Host Innate Immune Response to Viral and Bacterial Co-Infections

### Viral immunity

IAV infects both lung epithelial cells and alveolar macrophages, causing necrosis and apoptosis, respectively (81). Necrosis and apoptosis trigger the production of the transcription factor NF-κB, which regulates transcription of inflammatory genes such as tumor necrosis factor (TNF) α and IL-6, as well as chemokines such as monocyte chemoattractant protein-1 (81). These chemokines and cytokines cause infiltration of monocytes and neutrophils from the peripheral blood toward sites of infection. Multiple cycles of replication and infection lead to an exacerbated and unnecessarily inflamed innate immune response, leading to lung inflammation. Although a degree of inflammation is necessary for viral clearance, this exacerbation can lead to lethal lung injury. In fact, observations using C–C chemokine receptor (CCR) 5 and CCR2 knockout receptors indicate that the morbidity and mortality of influenza is not necessarily due to the viral load, but the innate immune response that follows (25). There is generally a correlation between disease severity and cytokine expression levels. In the case of high pathogenic disease, an excessive and uncontrolled expression of inflammatory cytokines, otherwise known as the “cytokine storm,” leads to excessive inflammation in the lung (81). Macrophages play a crucial role as the primary line of defense after influenza infection. Infected macrophages, despite undergoing apoptosis within 48 h, are able to produce large amounts of cytokines and chemokines, which contribute to the cytokine storm, leading to lung injury and edema (81).

### Bacterial immunity

Similar to the innate immune response against IAV, bacterial immunity is largely NF-κB dependent. Observations by Zhang *et al.* using antibodies against IL-17A saw a decrease in bacterial clearance associated with inadequate recruitment of monocytes and macrophages into the airway (187). Importantly, TNF-α plays a protective role in early infection and together with IL-1, it is responsible for neutrophil infiltration and cytokine expression, which allows for bacterial clearance (67). In addition, the complement system plays an important role in bacterial clearance, specifically in pneumococcal infections. Indeed, it was reported that loss of induction of the classical pathway in mouse models, involving genetic alteration of either C1q or immunoglobulin M, caused inadequate macrophage activation and progression of bacteremia (15). Innate cell immunity is highly dependent on the identification of pathogens, which occurs primarily through TLR activation.

### Toll-like receptors

TLRs are a class of pattern recognition receptors that detect pathogen-associated molecular patterns (28). TLRs are found on antigen-presenting cells such as dendritic cells, macrophages, and B cells. These receptors are critical in identifying pathogens, and therefore initiating the innate immune response, including cytokine and chemokine release (49). Ten human TLRs have thus far been identified, four of which, TLR3, TLR7, TLR8, and TLR9, are expressed within the endosome; whereas TLR4, TLR5, TLR2, and TLR2–6 are found on the cell surface (3, 70).

MyD88 is a critical adaptor protein used by all TLRs except for TLR3. MyD88 contains domains that allow its association with multiple proteins. The intermediate domain allows association with IL-1R-associated kinases (IRAKs) and a terminal domain that allows for TLR-TIR binding (59). TLR2 and TLR4 require the association of MyD88 to associate with TIR containing-domain adaptor protein (TIRAP) (56). Genetic knockouts in mice of TIRAP has led to an inability to produce cytokines on activation of TLR2 and TLR4, but not TLR7 or 9 (181). Downstream signaling from MyD88 leads to transduction of IRAKs, including IRAK1, IRAK2, IRAK3, IRAK4, and IRAKM, which all, either directly or indirectly, associate with TNF receptor-associated factor (TRAF) 6 (85, 161). TRAF6 regulates IκB kinases and MAPK pathways, in turn regulating NF-κB transcription factors (43, 84). In addition to NF-κB production, MyD88 is involved in the production of interferon regulatory factor 7 production, which promotes and activates the transcription of type I IFN (71). MyD88-independent pathways can be activated by TLR3 and TLR4 that utilize the TIR domain-containing adaptor inducing IFN-β to induce NF-κB and type I IFN production (65).

TLRs are able to maintain specificity to ligands yet target biologically conserved molecular patterns within their respective pathogens. TLR2 perhaps has the broadest range of ligands that can activate it, as it can form a heterodimer with TLR1 and TLR6 (37). A summary of the human TLRs is shown in Table 1.

**Table 1.** Summary of Known Human Toll-Like Receptors, Including Localization and Adaptor Proteins^a^

TLR	Target	Localization	Adaptor protein
TLR1	Triacyl lipopeptides	Cell surface	MyD88 TIRAP
TLR2	Glycolipids, lipopeptides, lipoproteins, lipoteichoic acid, β-glucan	Cell surface	MyD88 TIRAP
TLR3	Double stranded RNA	Intracellular compartmentalization	TRIF
TLR4	LPS	Cell surface	MyD88 TIRAP TRIF TRAM
TLR5	Flagellin	Cell surface	MyD88
TLR6	Diacyl lipopeptides	Cell surface	MyD88
TLR7	Single-stranded RNA	Intracellular compartmentalization	MyD88
TLR8	Single-stranded RNA	Intracellular compartmentalization	MyD88
TLR9	Double-stranded DNA	Intracellular compartmentalization	MyD88
TLR10	Triacyl lipopeptides	Cell surface	MyD88

^a^ Reference (169a).

LPS, lipopolysaccharide; MyD88, myeloid differentiation primary response 88; TIR, translocated intimin receptor; TLR, toll-like receptor; TRAM, translocating chain-associated membrane protein; TRAP, tryptophan-regulated attenuation protein; TRIF, TIR-domain-containing adapter-inducing interferon-β.

### NLRP3 inflammasome complex

Inflammasome complexes are multiprotein oligomers that form in response to danger signals and secrete the inflammatory cytokines IL-1β and IL-18. The diversity of the inflammasome NLRP3 has generated much interest as it can be stimulated by a range of pathogens, including, but not limited to, influenza and *Streptococcus* (163, 171). On receiving danger signals, the NLRP3 is associated with adaptor protein apoptosis-associated speck-like protein containing CARD, which contains a caspase recruitment domain. This complex binds to and activates caspase 1, which cleaves pro IL-1β and pro IL-18 into their activated forms IL-1β and IL-18, respectively (26, 94).

As NLRP3 can be activated to a range of stimuli, including sterile inflammatory disease, regulation is necessary to prevent autoimmunity and exacerbated inflammation. Multiple studies describe the dynamic role of IFN-β in the regulation of IL-1β and vice versa (57, 165, 166). Indeed, type I IFN knockout mice models showed increased production of IL-1 compared with their wild-type counterparts (17, 98). The regulation of IL-1β occurs at the NLRP3 inflammasome, where IFN-β induces inducible nitric oxide synthase (iNOS) expression. iNOS induces nitric oxide production, which inhibits the NLRP3 complex through S-nitrosylation (7, 52). IFN-β can also induce the anti-inflammatory cytokine IL-10 to downregulate IL-1β (80, 87). IL-1β inhibition of IFN-β is also believed to occur, however the mechanism behind it is not well established. Evidence suggests that IL-1β inhibits IFN-β-induced STAT1 activation by a proteasome-dependent mechanism to prevent phosphorylation (165). IL-1β can also generate prostaglandins to inhibit IFN-β expression (98). The dynamic interplay between IFN-β and IL-1β could provide a novel mechanism for the pathogenesis of influenza and bacterial co-infections. Classically, IFN-β and IL-1β are believed to be anti-viral and anti-bacterial cytokines, respectively. Influenza and bacterial pathogens may be taking advantage of these cytokines downregulating each other, preventing the effective clearance by the innate immune system.

### Trained immunity

Trained immunity is a rather novel innate immune mechanism. Until recently, innate immune cells were believed to retain no memory of infection; and that their response is generic and independent of whether they have been stimulated earlier. Indeed, only the adaptive arm of the immune response was believed to have immunological memory.

Trained immunity was first proposed in 1964, where on pathogens would confer resistance in host organisms against other, unrelated infections (90). This specific response was later attributed to IFN-γ production by memory T cells (91). Additional clues indicating a level of memory in innate immune cells stem from plants. Plants are able to retain an increased efficiency of pathogen resistance after repeated insults of unrelated infections (88). Indeed, it has been reported that this phenomenon is a form of epigenetic rewiring (88). Invertebrates have also been demonstrated to have a similar system of immunological memory. Lacking an adaptive immune response, invertebrates nonetheless displayed enhanced immunity against subsequent infection. One example showed bumblebees with stronger immunity and improved survival rates after secondary exposure to a lethal dose of plasmodium at 8 days compared with 22 days, regardless of whether the strains were related or not. The implication is that the innate immunity was imprinted with infection, and thus was primed and ready to more effectively deal with a secondary infection (143). At 22 days, it is likely that these innate cells underwent apoptosis and were replaced; therefore, macrophages had no prior activation and caused susceptibility to infection.

Vertebrates have also shown a nonspecific immunological memory. When mice were intravenously treated with β-glucan, a polypeptide found on the cell surface of fungi, mice had significantly increased survival rates post**–***S. aureus* challenge (29). Indeed, it has been described that the antibacterial action of β-glucan is independent of the Dectin-1 receptor, but likely due to a priming effect (93).

The mechanisms behind trained immunity remain relatively unknown. It may be plausible that prolonged activation of inflammatory pathways brings about epigenetic changes, leading to increased expression of TLRs, which can more readily and proactively react against invading pathogens (141). One such process could involve metabolic modifications within macrophages and natural killer cells. It is also possible that the inflammatory process and ROS production are different based on the infecting pathogen and there is competition between the mechanisms of innate and adaptive immunity.

### Immune tolerance

**FIG. 3.**
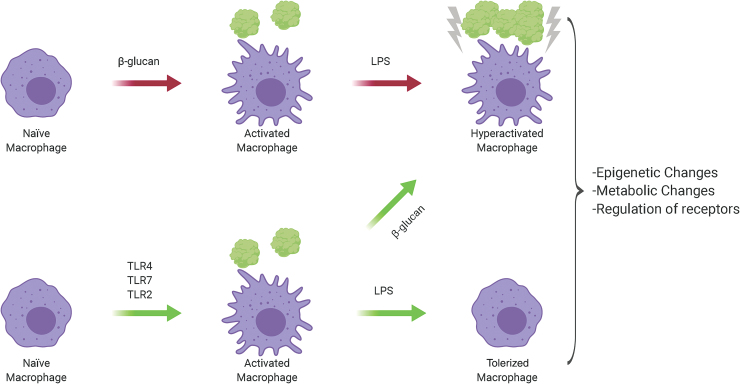
**Activation by either β-glucan or TLR4, 7 and 2 determines whether a macrophage will be “trained” or undergo tolerization.** These epigenetic changes include metabolic genes or could involve the regulation of receptors. β-Glucan can reverse the phenotype of a macrophage from tolerized to trained. Adapted from Novakovic *et al.* (119). TLR, toll-like receptor. Color images are available online.

Contrary to trained immunity, immune tolerance is the lack or significantly reduced activation of immune cells in response to a secondary stimulus. Classically, it is seen in the lack of a response in the adaptive immune system to self-antigens and non-harmful environmental stimuli such as allergens and gut microbes (Fig. 3).

Contrasting the role of β-glucan, innate immunity is also able to undergo immune tolerance, with the best described inducer being LPS (45, 92). Similar to trained immunity, these macrophages undergo epigenetic changes that involve altered metabolism, but this results in a significant reduction in cytokine and chemokine production compared with a classically activated macrophage (119). This is believed to occur as a safety countermeasure to the cytokine storm, and in the case of LPS, to limit septic shock (133). Multiple TLR receptors have been implicated in tolerance, among them being TLR4, TLR7, TLR2, and TLR5 (45, 74, 77, 159). These receptors are all involved in a bacterial/influenza co-infection, and thus the first pathogen to hit may inactivate the immune system, allowing the second to cause uncontrolled pathogenesis.

## Novel Pharmacological Strategies and Therapeutics

### SDH inhibitors

SDH, as previously mentioned, is a significant driver of mtROS production and is involved in the TCA and oxidative phosphorylation. Two classes of SDH inhibitors exist: One class targets the ubiquinone region, whereas the other targets the succinate region. Major applications of SDH inhibitors include commercial use as antifungals, as previously reviewed (155). Endogenous inhibitors of SDH include metabolite intermediates malate and oxaloacetate, as well as the synthetic compound malonate. In particular, oxaloacetate has been suggested to be a strong, irreversible inhibitor of SDH (Table 2). Cells pre-treated with oxaloacetate were unable to oxidize succinate, suggesting either tight association of oxaloacetate or conformational changes (185). Such inhibition is likely to occur to prevent RET, thus limiting ROS generation.

**Table 2.** List of Inhibitors Targeting Metabolic Pathways and Reactive Oxygen Species

Inhibitor	Function	Mechanism of action	Other information
2-Deoxy-d-glucose	Glycolysis inhibitor	Inhibits production of glucose-6-phosphate by irreversibly binding with hexokinase	Has been examined as a potential antitumor therapy (186). Unlikely feasibility due to cardiac side effects (111)
3-Bromopyruvate	Glycolysis inhibitor	Inhibits production of glucose-6-phosphate by irreversibly binding with hexokinase	Currently investigated as an anticancer agent (95)
Ritonavir	Glycolysis inhibitor	Inhibits the GLUT4 transport receptor	Currently used as part of antiretroviral therapy
Dichloroacetate	Glycolysis inhibitor	Inhibits pyruvate dehydrogenase kinase	Currently investigated as an anticancer agent (106)
FX11	Glycolysis inhibitor	Inhibits lactate dehydrogenase	
Rotenone	Electron transport chain inhibitor	Inhibits transfer of electrons in complex I from iron clusters, interfering with NADH synthesis. Ultimately causes a backflow of electrons leading to reverse electron transfer	Currently used as a pesticide
UK5099	TCA inhibitor	Pyruvate carrier inhibitor	Inhibits apoptosis
6-Aminonicotinamide	Pentose phosphate pathway inhibitor	Inhibits glucose-6-phosphate dehydrogenase	
Polydatin	Pentose phosphate pathway inhibitor	Inhibits glucose-6-phosphate dehydrogenase (104)	Has displayed anti-angiogenesis and proapoptotic properties
Bulthione Sulfoximine	Glutathione inhibitor	Inhibits gamma-glutamylcysteine synthetase	Currently investigated as an adjuvant in chemotherapy
MitoTEMPO	mtROS scavenger	Lipophilic cation group allows accumulation in the mitochondria, where the SOD mimetic piripidine nitroxide is able to scavenge superoxide	
MitoTEMPOL	mtROS scavenger	Similar to MitoTEMPO. Lipophilic cation group allows accumulation in the mitochondria, where the SOD mimetic piripidine nitroxide is able to scavenge superoxide	
MitoQuinone	mtROS scavenger	Similar to MitoTEMPO. Lipophilic cation group allows accumulation in the mitochondria. However, it has a quinone group that is able to scavenge two superoxide molecules	
Antimycin A	Electron transfer chain inhibitor	Inhibits oxidation of ubiquinol at cytochrome *C*, causing a backflow of electrons leading to reverse electron transfer	Currently used as a pesticide
Dimethyl itaconate	Cell-permeable itaconate derivative	Alkylates KEAP 1 to activate the antioxidant and anti-inflammatory gene nrf2 (110)	Can be metabolized and broken down to succinate (35). Rapidly degraded and unlikely to mimic itaconate
		Inhibits SDH	
4-Octyl itaconate	Cell-permeable itaconate derivative	Alkylates KEAP 1 to activate the antioxidant and anti-inflammatory gene nrf2 (110)	
		Inhibits SDH	
Malonate	Succinate dehydrogenase inhibitor	Competitively binds to the active site of succinate dehydrogenase	
Vitamin E	Antioxidant	Scavenges superoxide	Unreliable *in vivo* due to relatively slow reaction rate with superoxide. Would require very high intracellular doses that cannot be achieved
MitoVit-E	mtROS scavenger	Similar to Vitamin E, except contains triphenylphosphonium, which allows mitochondrial targeting. Inhibits lipid oxidation	
SkQ1	mtROS scavenger	Specific mechanism of action unknown	
Apocynin	NOX oxidase inhibitor and ROS scavenger	Reduces superoxide, prevents association of p47^phox^ with catalytic subunits	Commonly used in studies involving oxidative stress
Ebselen	ROS scavenger and NOX2 oxidase inhibitor	Glutathione peroxidase mimetic, converts hydrogen peroxide to water and oxygen. Also possesses NOX2 oxidase inhibitory activity	
Gp91ds-tat	NOX2 oxidase inhibitor	Inhibits association of p47^phox^ with the NOX2 subunit	
Cgp91ds-tat	Targeted endosomal NOX2 oxidase inhibitor	Inhibits association of p47^phox^ with the NOX2 subunit. Cholestanol conjugation allows for targeted delivery into the endosome through membrane anchoring	
GKT-831	NOX oxidase inhibitor	Dual NOX1 and NOX4 inhibitor. Specific mechanism of action unpublished likely due to ongoing clinical trials	Only antioxidant that has progressed and currently undergoing clinical trials
Diphenylene iodium	NOX oxidase inhibitor	Prevents electron flow from FAD through the flavocytochrome conduit	Commonly used in studies involving oxidative stress
ML090	NOX1 oxidase inhibitor	Mechanism of action unknown	
AEBSF	NOX2 oxidase inhibitor	Inhibits association of p47^phox^ with the NOX2 subunit	
Plumbagin	NOX4 oxidase scavenger	Mechanism of action unknown	Is known to have a wide range of therapeutic uses, including as an anti-inflammatory, anti-carcinogen, and anti-microbial

KEAP1, kelch-like ECH-associated protein 1; mtROS, mitochondrial ROS; Nrf2, nuclear erythroid factor 2; NOX, NADPH oxidase; ROS, reactive oxygen species; SDH, succinate dehydrogenase; SOD, superoxide dismutase; TCA, tricarboxylic acid.

Direct inhibition of SDH has been shown to have detrimental effects. Indeed, use of malonate caused neuronal legions similar to those seen in Parkinson's, Huntington's, and Alzheimer's disease (44). Further, genetic inhibition of the protein tumor necrosis factor type (tryptophan-regulated attenuation protein [TRAP]) 1, which chaperones SDH, appears to promote tumor development (48). However, it is important to note that inhibition of TRAP1 also exerted antioxidant and antiapoptotic effects (48). Thus, in the context of infections, acute inhibition of SDH can be explored as an avenue for ROS inhibition.

### Itaconate analogues

Recently, a potent, synthetic cell-permeable itaconate derivative, 4-octyl-itaconate (OI) was synthesized (Table 2). OI is different from a previous cell-permeable itaconate derivative, dimethyl itaconate (DMI), as DMI fails to be metabolized into itaconate; rather, it is metabolized into succinate (35). DMI is also a far more potent Nrf2 activator, as the lack of a negative charge leads to a push toward the Michael equation (14, 110) (Table 2). Thus, OI appears to be more reflective of endogenous itaconate; however, the relative novelty of OI results in a lack of understanding of its toxic properties.

### mtROS scavengers

Vitamin E was among the first pharmaceutical to show some activity against mtROS (96) (Table 2). Its efficacy, however, was undermined by multiple contributing factors. The first is that its reaction coefficient is far too slow to effectively compete with the conversion of superoxide to either H_2_O_2_ or peroxynitrite (76, 114). To effectively compete, a concentration of vitamin E is needed that is impossibly high to accomplish. The second factor is its non-specificity to the mitochondria, thus it fails to accumulate at the site required and contributes to the high concentration needed (164). Finally, vitamin E fails to cross the blood–brain barrier easily, and it can only do so when systemic concentrations are high for extended periods (16).

Mitoquinone (MitoQ) is a targeted mtROS scavenger, containing quinone enzymes, which allow for the uptake of two electrons (Table 2). MitoQ covalently bonds to the mitochondria through its lipophilic triphenylphosphonium cation, which accumulates in the mitochondria far more readily than the untargeted quinone scavenger. MitoQ protects against lipid peroxidation, by continuously scavenging superoxide and peroxynitrite, and is recycled back to quinone by SDH (72). Under certain conditions however, mitoQ can cause pro-oxidant effects, either by fuelling RET or by shifting to glycolysis over fatty acid oxidation, resulting in increased toxicity in the cell (38, 120). While having promising results in animal models, it is likely that these pro-oxidant effects nullified any therapeutic use of mitoQ in age-related skeletal muscle wasting and neurodegenerative diseases such as Parkinson's disease (102, 144, 157).

MitoTEMPO is another targeted mtROS scavenger, acting as an SOD mimetic (Table 2). MitoTEMPO specifically targets the mitochondria, leaving cytosolic and endosome ROS intact. Similar to mitoQ and the related compound MitoTEMPOL, MitoTEMPO also utilizes triphenylphosphonium to reach the mitochondria; however, it combines it with the antioxidant piripidine nitroxide (31). With a very low toxicity and relatively low IC50 concentration, MitoTEMPO is a strong candidate for mtROS-related diseases (115). Indeed, MitoTEMPO reduced diabetes-induced cardiomyocyte cell death and protected against paracetamol-induced hepatotoxicity (33, 116). MitoTEMPO also showed increased cell viability and reduction in inflammatory markers within the endothelium in preeclampsia models (100). In response to sepsis, MitoTEMPO has had both positive and negative success. Acute kidney injury models of sepsis showed a significant survival improvement, from baseline survival rate to 80% with MitoTEMPO (132). Conversely, a cecal ligation and puncture model of sepsis showed no improvement in survival rate (136). There may be reasons as to why these differences in the efficacy of MitoTEMPO arise. First, Rademann *et al.* (136) use a chronic model of sepsis, which may allow for the cell to recycle the accumulated MitoTEMPO in a similar manner as it recycles mitoQ. Second, the procedure may have induced injury that is not mtROS related and thus produced similar levels of inflammation and lethality. Lastly, it is likely that the body utilizes mtROS as an inflammatory signaling molecule against incoming microbes from food, and thus targeting the mtROS in the gastrointestinal tract is counterproductive as confounding pathogens may interfere. Within the respiratory system, MitoTEMPO has not adequately been explored as a therapeutic against pathogens but remains a viable option.

### NOX inhibitors

Due to the clinical relevance of NOX2, several inhibitors have been developed for experimental use. Although some of these have shown promising results both *in vivo* and *in vitro*, they remain unable to be translated to the clinical setting because of their pharmacology. These inhibitors have been reviewed (12, 18, 32, 47, 64, 82, 149, 151, 175) in the context of chronic diseases such as CVD, cancer, and neuronal diseases.

Multiple NOX inhibitors are presently used in experimental work, however in terms of infectious diseases perhaps the best described are apocynin and gp91ds-tat (or NOX2ds-tat). Apocynin is a pro-drug that is activated *via* dimerization in the presence of MPO to function as a dual NOX inhibitor and an antioxidant, preventing the association of p47^phox^ with the NOX2 subunit (53) (Table 2). Indeed, *in vitro* administration of apocynin showed a reduction in cytokines IL-6, IFN-β, CXCL10, and CCL5, likely as a consequence of the increased expression of the inflammatory cytokine modulators SOCS1 and SOCS3, in low, moderate, and high pathogenic strains of influenza (183). *In vivo* using a mouse model administration of apocynin significantly suppressed viral titer, airway inflammation, and inflammatory cell superoxide production after infection with X-31 or PR8 IAV (176). In IAV-induced exacerbations of acute cigarette smoke-induced lung disease, apocynin was able to decrease the presence of inflammatory BALF cells, and cytokine and chemokine expression in infected mice compared with their uninfected counterparts at days 3 and 7 post-infection, with no effect on the viral load (123). Ebselen, a Gpx mimetic and an NOX2 oxidase inhibitor, also reduced inflammatory cells in the BALF, and cytokine and chemokine production in cigarette smoke-exposed mice, in addition to reducing the viral load (123) (Table 2). The inability of apocynin to alleviate viral burden is interesting, as a separate study reported a reduction in viral titers caused by apocynin (176). These differences in viral suppression might be due to differences in dose. In Oostwoud *et al.* (123), a dose of 5 mg/kg was used compared with 2.5 mg/kg used in Vlahos *et al*. (176), potentially having off-target effects such as NOX1, which has been shown to be protective in IAV infections (150). Interestingly, apocynin was able to increase the efficacy of the antibiotic linezolid against a methicillin-resistant *S. aureus* coinfection *in vivo* (160). Thus, this indicates that despite the critical role that NOX2 plays in the clearance of bacterial pathogens, an enhanced NOX2 activity by IAV infection yields no improvement in outcome, but rather contributes to the pathology and hampers pharmacological intervention.

Gp91ds-tat is a peptide inhibitor of NOX2, containing a sequence related to the gp91^phox^ subunit, effectively preventing the binding of the NOX2 subunit with p47^phox^ (139) (Table 2). The sequence is also linked to the tat peptide derived from human immunodeficiency virus, which facilitates entry into the cell. Gp91ds-tat has shown a reduction in inflammatory cells in the BALF during influenza infections (169). Further, when Gp91ds-tat was conjugated to cholestenol and a polyethylene glycol-linker to specifically target endosomal NOX2, there were further decreases in airway inflammation, as well as elevations in the anti-viral cytokine IFN-β (169) (Table 2). Cholestanol-conjugated gp91ds-TAT was very effective at preventing severe lung inflammation to the highly pathogenic IAV PR8 infection in a mouse model. It resulted in a substantial suppression of neutrophil infiltration and the alveolitis to PR8 infection in mice (168).

## Concluding Remarks

**FIG. 4.**
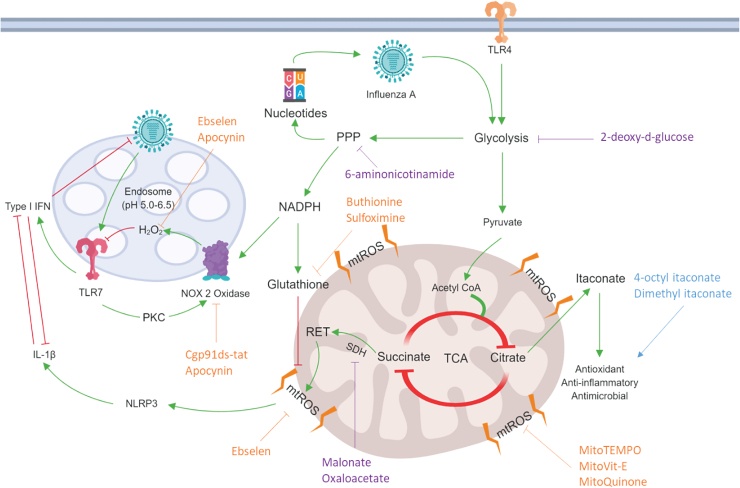
**Therapeutic targets of immunometabolism and ROS against influenza.** Influenza is believed to cause a metabolic switch, which upregulates glycolysis and the PPP through a breakdown of the TCA cycle. As a by-product, nucleotides from the PPP form the building blocks for a new virus. NADPH can be used for glutathione production as an antioxidant. Conversely, NADPH can also be used to fuel NOX2 oxidase, which is in its own intricate, negative feedback loop with TLR7 and the virus (169). ROS inhibitors, seen in *orange*, can be employed to target specific sites, which can alleviate oxidative stress by inhibiting NOX2, mtROS production, or scavenging ROS. Alternatively, therapeutic strategies could include inhibition of metabolic pathways, seen in *purple*, to shunt viral reproduction and inflammatory pathways through glycolysis (109, 162), PPP inhibition, and SDH (2-deoxy-d-glucose, 6 aminonicotinamide, and malonate and oxaloacetate, respectively). Itaconate analogues, seen in *blue*, are proposed to increase therapeutic outcomes in response to infection; through increased activity of Nrf2 to upregulate crucial antioxidant genes, to direct antimicrobial and anti-inflammatory effects (110). mtROS, mitochondrial ROS; Nrf2, nuclear erythroid factor 2; SDH, succinate dehydrogenase. Color images are available online.

With viral and bacterial infections becoming more resistant to antimicrobial therapies, there is an ever growing need to develop cost-effective strategies that will ease the health care burden. This review discussed multiple, distinct but interconnected redox-dependent and immunometabolic mechanisms for which the enhanced pathogenesis of influenza and bacterial co-infections may arise, and where future therapeutics could be developed (Fig. 4). Indeed, a greater understanding of trained immunity in a co-infection model may result in harnessing appropriate interventions, including re-adjusting the metabolic switch to establish an optimum level of activation for macrophages while limiting excessive inflammation. Indeed, early promising results in unrelated LPS-induced sepsis models (128) gives rise to new and exciting approaches to lower respiratory tract infections.

## Abbreviations Used

AKT: protein kinase B
AMPK: 5′ adenosine monophosphate-activated protein kinase
ATP: adenosine triphosphate
BALF: bronchoalveolar lavage fluid
CCR: C–C chemokine receptor
CD: cluster of differentiation
Cgp91ds-tat: cholestenol-conjugated gp91ds-tat
COPD: chronic obstructive pulmonary disease
CVD: cardiovascular disease
CXCL: C–X–C ligand
DMI: dimethyl itaconate
DUOX: dual oxidase
FADH_2_: flavin adenine dinucleotide
Gpx: glutathione peroxidase
GSH: glutathione
H1N1: hemagglutinin 1 neuraminidase 1
H_2_O_2_: hydrogen peroxide
HIF: hypoxia inducible factor
Hk X-31: Hong Kong strain X-31
HMOX1: heme oxygenase
IAV: influenza A virus
IFN: interferon
IL: interleukin
iNOS: inducible nitric oxide synthase
IRAK: IL-1R-associated kinase
IRG1: immune responsive gene 1
KEAP1: kelch-like ECH-associated protein 1
LPS: lipopolysaccharide
MAPK: mitogen-activated protein kinase
MIP: macrophage inflammatory protein
MitoQ: mitoquinone
MRSA: methicillin-resistant *Staphylococcus aureus*
mtROS: mitochondrial ROS
MyD88: myeloid differentiation primary response 88
NADH: nicotinamide adenine dinucleotide
NADPH: nicotinamide adenine dinucleotide phosphate
NET: neutrophilic extracellular trap
NF-κB: nuclear factor kappa B
NLRP: nod-like receptor protein
NO: nitric oxide
NOX: NADPH oxidase
NRF2: nuclear factor erythroid 2
OI: 4-octyl-itaconate
PI3K: phosphoinositide 3-kinase
PPP: pentose phosphate pathway
PR8: Puerto Rico strain of IAV
PspA: pneumococcal surface protein A
PsrP: pneumococcal serine-rich repeat protein
RET: reverse electron transfer
ROS: reactive oxygen species
SDH: succinate dehydrogenase
SOD: superoxide dismutase
STAT: signal transducer and activator of transcription
TCA: tricarboxylic acid
TIR: translocated intimin receptor
TIRAP: TIR containing-domain adaptor protein
TLR: toll-like receptor
TNF: tumor necrosis factor
TRAF: TNF receptor-associated factor
TRAM: translocating chain-associated membrane protein
TRAP: tryptophan Regulated Attenuation Protein
TRIF: TIR-domain-containing adapter-inducing interferon-β
VEGF: vascular endothelial growth factor
VEGFR: vascular endothelial growth factor receptor
